# Association between plasma proteome and pulmonary heart disease: A two‐stage Mendelian randomization analysis

**DOI:** 10.1111/crj.13775

**Published:** 2024-06-03

**Authors:** Shiyang Li, Haifeng Ding, Qi Li, Xiaobin Zeng, Yanyu Zhang, Chengyi Lai, Xiaoshuang Xie, Yongjiang Tang, Jianjun Lan

**Affiliations:** ^1^ Division of Cardiology Panzhihua Central Hospital Panzhihua China; ^2^ Dali University Dali China; ^3^ Department of Genealogy Panzhihua Central Hospital Panzhihua China; ^4^ Division of Cardiology The First Affiliated Hospital of Shihezi University Shihezi China; ^5^ Clinical Laboratory Center Panzhihua Central Hospital Panzhihua China; ^6^ Department of Vascular Diseases Panzhihua Central Hospital Panzhihua China

**Keywords:** *CAMK1*, Mendelian randomization, plasma proteome, pulmonary heart disease

## Abstract

Pulmonary heart disease (PHD) involves altered structure and function of the right ventricle caused by an abnormal respiratory system that causes pulmonary hypertension. However, the association between changes in plasma proteomics and PHD remains unclear. Hence, we aimed to identify causal associations between genetically predicted plasma protein levels and PHD. Mendelian randomization was performed to test the target proteins associated with PHD. Summary statistics for the human plasma proteome and pulmonary heart disease were acquired from the UK Biobank (6038 cases and 426 977 controls) and the FinnGen study (6753 cases and 302 401 controls). Publicly available pQTLs datasets for human plasma proteins were obtained from a largescale genome‐wide association study in the INTERVAL study. The results were validated using a case–control cohort. We first enrolled 3622 plasma proteins with conditionally independent genetic variants; three proteins (histo‐blood group ABO system transferase, activating signal cointegration 1 complex subunit 1, and calcium/calmodulin‐dependent protein kinase I [CAMK1]) were significantly associated with the risk of pulmonary heart disease in the UK Biobank cohort. Only CAMK1 was successfully replicated (odds ratio: 1.1056, 95% confidence interval: 1.019–1.095, *p* = 0.0029) in the FinnGen population. In addition, the level of CAMK1 in 40 patients with PHD was significantly higher (*p* = 0.023) than that in the control group. This work proposes that CAMK1 is associated with PHD, underscoring the importance of the calcium signaling pathway in the pathophysiology to improve therapies for PHD.

## Graphical abstract

Mendelian randomization (MR) was performed to test the target protein from the UK Biobank and the FinnGen study is associated with pulmonary heart disease. Calcium/calmodulin‐dependent protein kinase I (*CAMK1*) is associated with pulmonary heart disease.

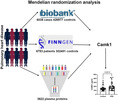

**Abbreviation**
PHD: pulmonary heart disease
MR: Mendelian randomization
SNPs: single nucleotide polymorphisms
CAMK1: calcium/calmodulin‐dependent protein kinase I
PH: pulmonary hypertension
RCT: randomized controlled trial
IVW: inverse variance weighted
CFTR: CF transmembrane conductance regulator
GWAS: genome‐wide association study
PPAR‐γ: peroxisome proliferator‐activated receptors‐γ
PASMC: pulmonary arterial smooth muscle cell
MLCK: myosin light streptokinase

## 1 INTRODUCTION

Pulmonary heart disease (PHD), also called Cor pulmonale, can be defined as hypertrophic remodeling and impaired function of the right ventricle and is nearly always caused by pulmonary hypertension (PH), which is usually secondary to chronic obstructive pulmonary disease.^1^ Overall, PH affects approximately 11–26 cases per million adults,^2^ and over half of the patients with heart failure may be affected.^3^ Although the right heart catheter is the gold standard, Doppler echocardiography is presently based on the noninvasive diagnosis of PH. However, until now, early detection treatment options for PHD have been limited, and the five‐year survival rate of patients with chronic obstructive pulmonary disease having PH is approximately 50%.^4^ By genetic approaches, multiple lines of evidence have discovered variants of *BMPR2* (NG_009363.1), *SMAD1* (NG_042284.1), *SMAD4* (KF572433.1), *SMAD9* (NG_016963.1), and other genes linked to the development PH.^5^, ^6^, ^7^ However, researchers have not paid proper attention to dysregulated gene expression in PHD. Several conventional drugs prevent the development of PHD. Therefore, it is important to identify the relation between genetically predicted plasma protein levels and PHD.

Mendelian randomization (MR) uses common variants as independent proxies for the exposure of interest to investigate causal associations by mimicking a randomized controlled trial with SNP as instrumental variables. Some MR analyses have demonstrated that the CF transmembrane conductance regulator is associated with PH risk.^8^ Magnetized target by MR analysis has identified interleukin‐6 as a drug target in pulmonary arterial hypertension (PAH).^9^ In addition, the whole‐blood RNA profiles were associated with PAH.^10^ Additionally, an imbalance in the circulating proteome is an early hallmark of the disease as a result of dynamic assembly in the influx and efflux of proteins synthesized by tissues and cells across the body. Using genetic markers, the plasma proteome of PAH confirmed that two proteins, netrin‐4 and thrombospondin‐2, are associated with pulmonary arterial pressure.^11^ Moreover, serum proteome profiling has shown that Zhang^12^ is a promising biomarker of chronic thromboembolic PH. These studies discussed new biomarkers of PH using the plasma proteome.

However, genomic evidence of the plasma proteome in PHD has not yet been obtained. In the current study, we aimed to investigate the plasma proteome of patients with PHD and performed MR analyses by combining pQTL found in the blood with two independent PHD genome‐wide association study (GWAS) datasets. From the 3622 plasma proteins, we chose those that had significantly different circulating levels in patients with PHD compared with those in healthy controls. By measuring these data with MR, we identified CAMK1 (EF444963.1) in the calcium signaling pathway in the pathobiology of PHD.

## 2 METHOD

### 2.1 Summary statistics data for human plasma proteome and PHD

Publicly available pQTLs datasets for human plasma proteins were obtained from a large‐scale GWAS by Sun et al, who measured 3622 plasma proteins in 3301 healthy European blood donors from the INTERVAL study using an aptamer‐based multiplex protein assay (SOMAscan).^13^

PHD datasets were downloaded from the UK Biobank Study and FinnGen Consortium (Table 1). The UK Biobank is a large‐scale biomedical database and a research resource containing in‐depth genetic and health information. The summary statistical data used in our study comprised 6038 cases and 426 977 controls, and the GWAS results were adjusted for age, sex, and 10 genetic principal components.

**TABLE 1:** The summary of datasets in our study.

Triats	Data source/PMID	Population	Sample size	Case	Control	Covariates adjusted in GWAS
Plasma proteome	29 875 488	European	3301	‐	‐	Age, sex, duration between blood draw and processing (binary, ≤1 day/>1 day) and the first three principal components of ancestry
Pulmonary heart disease	The UK Biobank study (UKBB)	European	433 015	6038	426 977	Age, sex, and 10 genetic principal components
	The FinnGen consortium	European	309 154	6753	302 401	Age, sex, the first 10 genetic principal components, and genotyping batch

**FIGURE 1:**
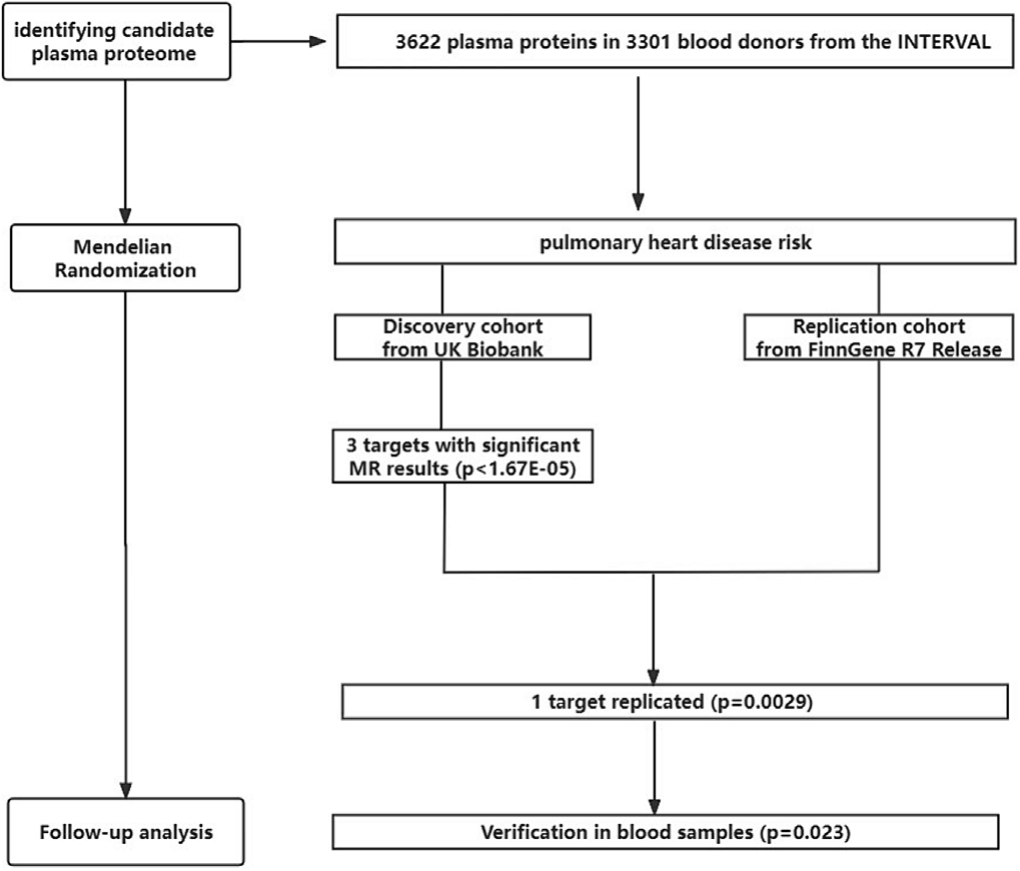
The flowchart of the study.

The replication dataset for PHD was obtained from the FinnGen consortium R7 release, comprising 6753 cases and 302 401 controls. Several covariates were also adjusted for, including age, sex, the first 10 genetic principal components, and the genotyping batch. The flowchart is shown in Figure 1.

### 2.2 Selection of instrumental variables for plasma proteins

We identified SNPs that were associated with a significant plasma protein at the threshold of *p* < 1 × 10^−5^ but not associated with PHD (*p* < 5 × 10^−8^). Linkage disequilibrium among SNPs for one exposure was estimated using the PLINK clumping method based on the 1000 Genomes European reference panel, and independent SNPs without linkage disequilibrium (r^2^ < 0.01, clump window > 10 kb) were used as instrumental variables. Additionally, all SNPs used in the MR analysis should be sufficiently strong; therefore, an F‐statistic >10 was considered sufficiently informative.

### 2.3 Statistical analysis

All analyses were performed using the TwoSampleMR package in the R Software 3.6.1.^14^ Single‐SNP MR estimates were calculated using Wald ratios. For multiple SNP variables, we used an inverse variance‐weighted (IVW) model to derive a combined causal estimate.^15^ Odds ratios (ORs) and 95% confidence intervals (CIs). Several sensitivity analyses were conducted to evaluate the robustness of causality and to detect pleiotropy. The weighted median method provided a consistent estimate if less than 50% of SNPs were invalid instruments.^16^ The MR‐Egger method provides valid MR estimates in the presence of horizontal pleiotropy when the pleiotropic effects of the genetic variants are independent of genetic associations with exposure.^17^ Furthermore, we tested for pleiotropy using the MR‐Egger regression intercept and Cochran's Q test. Considering the potential false positive rate, we set a significance threshold of 0.05/2987 = 1.67 × 10^−5^ in the discovery stage and 0.05/3 = 0.0167 as significant in the replicate analysis. The diagnostic performance of the model was measured using the area under the curve (AUC) of the receiver operating characteristic (ROC) curve, and all models included CAMK1 levels and common clinical risk factors (age, TG, LDL, HDL, GLU, EF, and RVED). Correlation analysis was conducted using the Spearman's method.

### 2.4 Human participants

We enrolled 80 patients from Panzhihua Central Hospital, Panzhihua, Sichuan. Between January and December 2022, 40 patients with PHD were diagnosed using Doppler ultrasound or computed tomography, along with 40 matched healthy control participants (Table S4). The blood samples were separated immediately by centrifugation and stored at −80°C.

### 2.5 Enzyme‐linked immunosorbent assay (ELISA) analysis of CAMK1

To test the levels of calcium/calmodulin‐dependent protein kinase I (CAMK1) in the plasma of PHD mice, we used a Human *CAMK1* (EF444963.1) ELISA Kit (Themofisher scientific, MA5‐43469) according to the manufacturer's protocol.

## 3 RESULTS

### 3.1 Study design

To investigate causal associations between genetically predicted plasma protein levels and PHD, we conducted a two‐stage MR analysis. First, we selected conditionally independent genetic variants associated with plasma protein levels. Subsequently, we used the UK Biobank cohort as the discovery cohort and conducted a two‐sample MR analysis. After harmonizing the exposure and outcome datasets and removing palindromic SNPs, 2987 plasma proteins remained to be explored further. Statistically significant associations determined using the Bonferroni method were replicated in the FinnGen consortium population.

### 3.2 Causal estimates of genetically predicted proteins on PHD

**FIGURE 2:**
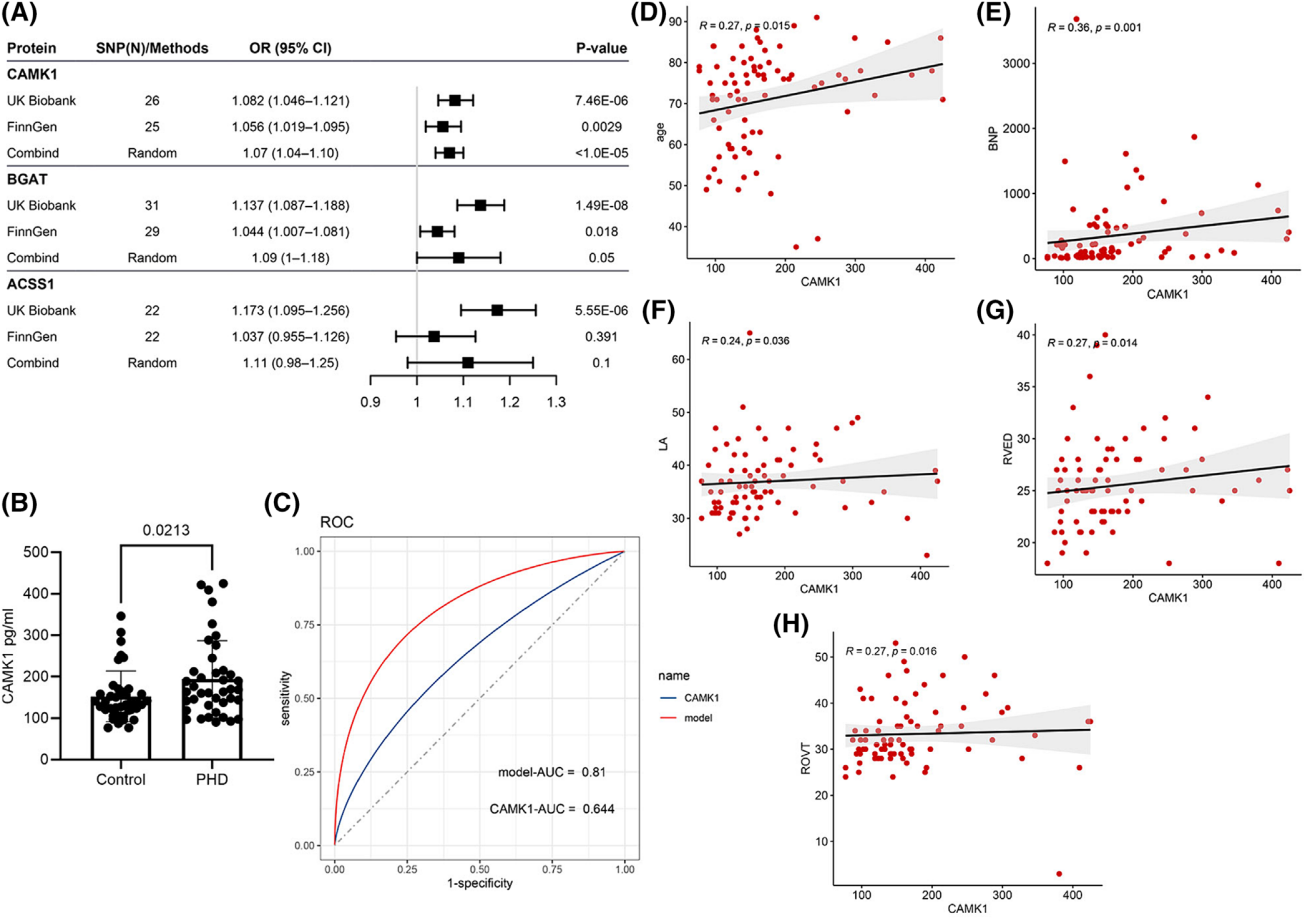
(A) Forest plot showed genetically‐predicted plasma proteome of *CAMK1* is associated with pulmonary heart disease risk. (B) *CAMK1* expression levels in peripheral blood lymphocytes were comprised of control and PHDs. (C) The AUC of the CAMK1 level was 0.644, when added age, TG, LDL, HDL, GLU, EF, and RVED into the model, the AUC of the ROC was 0.81 (Figure 2C). *CAMK1* concentrations have positive related to age, BNP, LA, RVED, and ROVT (Figure 2D–H).

In the UK Biobank cohort, genetically determined expression of three proteins was significantly associated with the risk of PHD at *p* < 1.67 × 10^−5^, including histo‐blood group ABO system transferase (BGAT NC_060936.1, OR: 1.137, 95% CI: 1.087–1.188, *p* = 1.49 × 10^−8^), activating signal cointegration 1 complex subunit 1 (ACSS1 NC_060944.1, OR: 1.173, 95% CI: 1.095–1.256, *p* = 5.55 × 10^−6^), and calcium/calmodulin‐dependent protein kinase type 1 (CAMK1, OR: 1.082, 95% CI: 1.046–1.121, *p* = 7.46 × 10^−6^) (Figure 2A, Table S1). Moreover, there was weak evidence for associations between genetically predicted 159 protein levels and PHD (1.67 × 10^−5^ < *p* < 0.05).

In the FinnGen population, only CAMK1 was successfully replicated (OR: 1.1056, 95% CI: 1.019–1.095, *p* = 0.0029) (*p* < 0.0167), but there was limited evidence supporting the association of genetically proxied BGAT levels with PHD (OR: 1.044, 95% CI: 1.007–1.081, *p* = 0.018). The combined analysis using the random effects model indicated CAMK1 (OR: 1.07, 95% CI: 1.04–1.10, *p* < 1.0 × 10^−5^) remains to be significant, but not BGAT (OR: 1.09, 95% CI: 1.0–1.18, *p* = 0.05). Furthermore, 10 proteins were weakly replicated among the 159 proteins in the UK Biobank (Table 2).

**TABLE 2:** The suggested an association between plasma proteins and pulmonary heart disease.

Protein	Method	UK Biobank dadaset			FinnGen dadaset		
		NSNP	OR (95% CI)	*p*‐value	NSNP	OR (95% CI)	*p*‐value
Clusterin	Inverse variance weighted	18	0.897 (0.829–0.971)	0.007	18	1.08 (1.004–1.162)	0.039
Vesicle‐fusing ATPase	Inverse variance weighted	20	0.893 (0.819–0.973)	0.01	19	0.903 (0.836–0.974)	0.009
Complement C1q‐like protein 4	Inverse variance weighted	16	0.898 (0.825–0.977)	0.012	15	1.104 (1.022–1.193)	0.012
Interleukin‐36 beta	Inverse variance weighted	22	1.093 (1.018–1.173)	0.014	20	1.079 (1.011–1.152)	0.022
Neuroendocrine convertase 1	Inverse variance weighted	33	0.961 (0.929–0.995)	0.025	33	0.968 (0.938–0.998)	0.039
UPF0454 protein C12orf49	Inverse variance weighted	18	0.915 (0.844–0.992)	0.03	16	1.103 (1.013–1.2)	0.024
Creatine kinase B‐type	Inverse variance weighted	27	0.933 (0.873–0.997)	0.04	23	1.081 (1.012–1.154)	0.02
Calcium/calmodulin‐dependent protein kinase type II subunit beta	Inverse variance weighted	11	1.121 (1.003–1.252)	0.044	11	1.11 (1.002–1.231)	0.046
Synaptotagmin‐3	Inverse variance weighted	11	1.111 (1.002–1.231)	0.045	11	0.915 (0.838–1)	0.049
Alpha‐(1,6)‐fucosyltransferase	Inverse variance weighted	29	0.963 (0.927–0.999)	0.046	28	0.961 (0.928–0.996)	0.03

### 3.3 Horizontal pleiotropy sensitivity analysis

**FIGURE 3:**
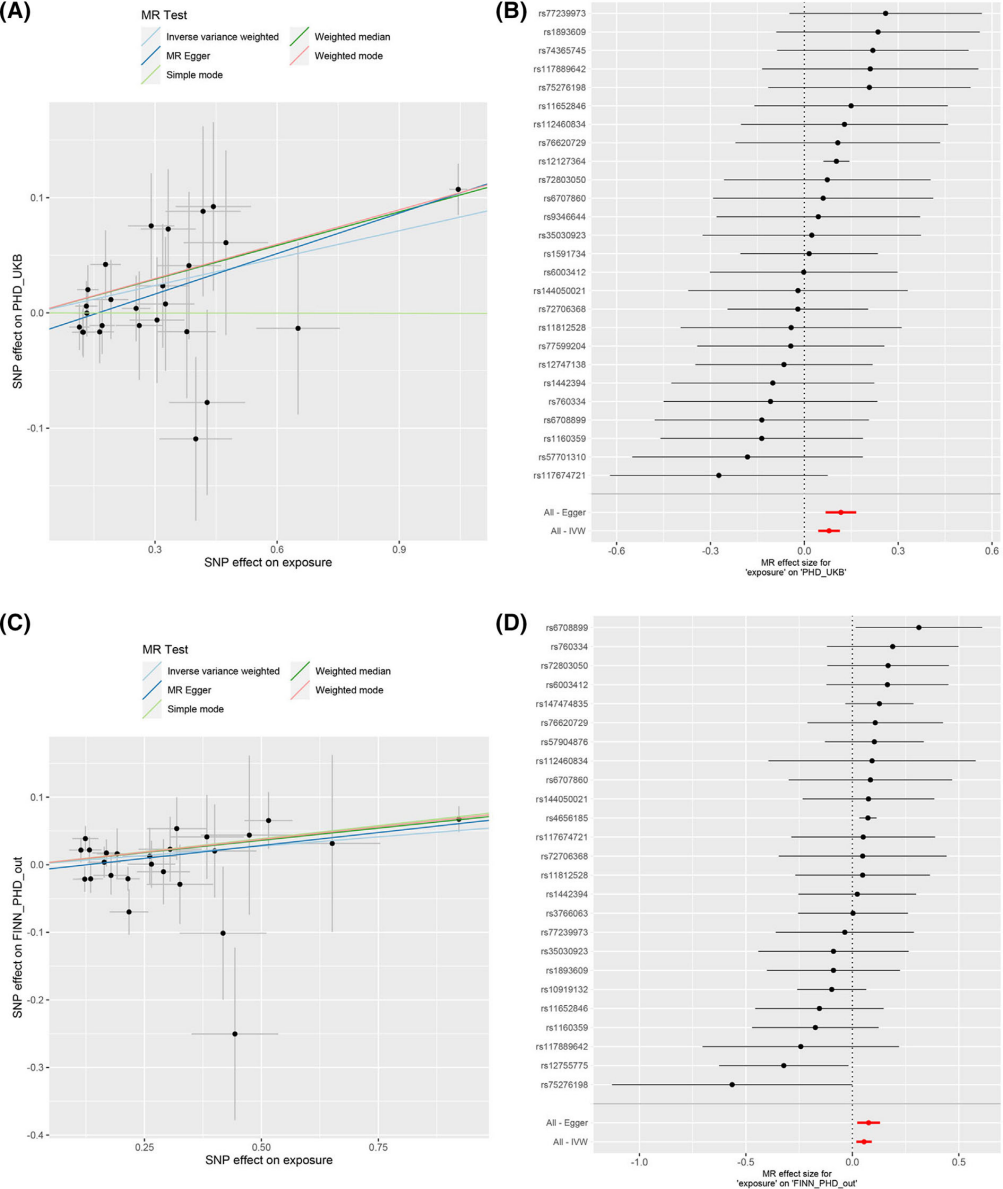
Scatter plot and forest plot of causal estimates of plasma proteome of *CAMK1* in pulmonary heart disease in UKB (A, B) and FINN (C, D).

We demonstrated that genetically predicted CAMK1 was related to the risk of PHD using the IVW method, and the results remained consistent in both magnitude and direction using the weighted median and MR‐Egger approaches (Table S2). Overall, our results showed little evidence of heterogeneity in the effects of genetic instruments (Figure 3). Although the MR‐Egger intercept *p* was 0.043 in the UK Biobank dataset, no obvious heterogeneity was observed in Cochrans Q statistic (Q‐IVW = 21.03, *p* = 0.69, Table S3). We also did not find any apparent horizontal pleiotropy, as indicated by the MR Egger intercepts, where all *p*‐values for intercepts were greater than 0.05 in the FinnGen datasets (Table S3).

In addition, to explore the possible relation between *CAMK1* and PHD risk, we detected the protein levels of blood samples in patients with PHD and controls and found that the expression of *CAMK1* was significantly upregulated in patients (*p* = 0.023, Figure 2B). The AUC of the ROC was 0.644, the sensitivity was 60%, and the specificity was 75% for the CAMK1 level; the AUC of the ROC was 0.81 when age, TG, LDL, HDL, GLU, EF, and RVED were added into the model (Figure 2C). Additionally, we found that the CAMK1 concentration was significantly positively correlated with age, BNP, LA, RVED, and ROVT (Figure 2D–H).

## 4 DISCUSSION

The present study investigated the causal association between the plasma proteome and PHD using MR methods by integrating GWAS datasets and gene expression data (eQTL and pQTL). This analysis used 12 791 patients with PHD and 729 378 control individuals. Our MR analysis showed evidence supporting that CAMK1 was significantly associated with the risk of PHD in the UK Biobank cohort and FinnGen populations. Furthermore, we detected the level of CAMK1 by ELISA in the study and verified the same results. Interestingly, few studies support the association between *CAMK1* and the risk of PHD.

The irreversible nature of PH advances pulmonary vascular obliteration and right ventricular dilation, caused by the cor pulmonale. PH is a key component of the pathophysiology of right ventricular dysfunction.^1^ Several studies have explored the causal association between PH and risk factors using MR. Zhang^18^ reported that IL‐6 signaling reduces the risk of PAH using IVW (OR = 0.023, 95% CI: 0.0013–0.393; *p* = 0.0093), which could be a drug target in PAH.^9^ Mining the plasma proteome of PAH revealed that two secreted matrix‐binding proteins (netrin‐4 and thrombospondin‐2) participate in the pathobiology of PAH.^11^ Although these studies have probed the factors of PAH, the association between the plasma proteome and PHD using MR has rarely been reported.

Additionally, proteins circulating in the blood offer many opportunities to diagnose or prevent diseases and have become an important tool for exploring the human morbid state. Li^19^ employed proteome‐wide MR to identify causal plasma proteins in venous thromboembolism development and explored the association between the plasma proteome and MR. We discuss examples in which plasma proteomics have contributed valuable insights into PHD and the opportunities offered by combining proteomics with genetic data. Here, we show that CAMK1 is associated with PHD using MR analysis, which may offer novel technological developments and possible clinical applications for PHD. In our study, we also reported the differences in the concentrations of CAMK1 in PHD and control cases using ELISA. A previous study showed that CAMK1 was highly expressed in pancreatic cancer^20^ and hepatocellular carcinoma.^21^ *CAMKI* is widely expressed in most mammalian cells and participates in many cellular functions, including nerve conduction, growth, aldosterone expression, and the cell cycle.^22^ However, the mechanisms and functions of *CAMK1* involved in cardiopulmonary diseases remain unclear.

Due to its excitation‐contraction coupling function, calcium plays an important role in the cardiovascular system. Studies have reported a link between the calcium signaling pathway and PH. RV samples from patients with PAH have shown that Ca^2+^ handling proteins contribute to RV diastolic dysfunction. In vitro and in vivo experiments have revealed that the Ca^2+^‐activated chloride channel ANO1/TMEM16A affects smooth muscle contraction and sensory signal transduction.^23^ Ca^2+^/*CAMK1* and peroxisome proliferator‐activated receptors‐γ are involved in PH, and overexpression of miR‐21 inhibited the expression of *CAMK1* and peroxisome proliferator‐activated receptors‐γ in pulmonary arterial smooth muscle cells.^24^

Although the study concluded an association between *CAMK1* and remodeling in pulmonary arteries,^25^ no study has been conducted to explore the effect of *CAMK1* after right heart failure. The current study reported that *CAMK1* is involved in the entire process of disease progression from PH to PHD. There are several possible explanations for the association. First, calcium is key to regulating the vascular luminal diameter and blood flow. *CAMK1* may activate myosin light chain kinase, leading to the phosphorylation and contraction of myosin and increasing pulmonary vascular resistance.^26^ Moreover, hypoxia induces an increase in apoptosis by intracellular Ca^2+^ overload,^27^ and the Ca^2+^/CaM‐dependent kinase signaling cascade could be enhanced by *CAMK1*, to accelerate the transduction of the Ca^2+^ signal.^28^ In addition, interactions with a short sequence of *CAMK1* regulatory domain, CaM, directly activate *CAMK1* as well.^29^ Second, *CAMK1* may regulate inflammation.^30^ However, its function in the pulmonary vasculature has not yet been discussed. Third, the heart's primary function is to conduct the myocardial cardiac cycle as well as systemic and pulmonary circulation. Ca^2+^ signaling interacts with many physiological processes. CaM (including *CAMK1*) influences Ca^2+^, particularly those that regulate cAMP and cGMP signaling. Ca^2+^ overload is a major cause of cellular injury during myocardial ischemia/reperfusion. Hyperactivation of *CAMK1* accentuates the effort.^31^ Furthermore, research has shown that *CAMK1* expression was significantly downregulated in patients with atrial myocyte hypertrophy.^32^ Although there is insufficient scientific evidence to prove *CAMK1* is involved in the development of right‐sided heart failure, direct or indirect activation of upstream and downstream genes of *CAMK1* regulates Ca^2+^ signals related to their different functions in the myocardium.

This study has several strengths. First, summary statistics of the two subtypes of PHD were obtained to calculate the causal association between the plasma proteome and PHD. We combined proteomics with genetic data to improve the efficacy and risk stratification of PHDs and found that one target (CAMK1) was associated with PHDs. Second, we performed an MR analysis to identify potential safety aspects and alternative indications that might be used in further clinical studies. Third, we determined the concentration of *CAMK1* in a population‐based, small‐sample study.

This study has several limitations. First, the potential influence of directional pleiotropy results in biased MR results, which is difficult to completely exclude from this study. Second, the effect observed in our study was a confined association between the plasma proteome and PHD, and more attention should be paid to the mechanism of *CAMK1* in the future. Third, further clinical research is required to evaluate the efficacy of managing PHDs. Fourth, the study samples were of European ancestry, and the insight of the study needs further experiments to confirm its applicability to other ethnicities.

In conclusion, this study indicated that *CAMK1* is a risk factor for PHD. However, randomized trials need to be conducted in the future to evaluate the causality of the PHD.

## AUTHOR CONTRIBUTIONS

Shiyang Li developed the study concept, designed and interpreted the data, and drafted the manuscript. Qi Li and Yanyu Zhang performed the research, Haifeng Ding undertaken the statistical analysis; Xiaobin Zeng, Chengyi Lai, and Xiaoshuang Xie extracted the information from the databases; and Yongjiang Tang and Jianjun Lan supervised the design of the study and revised the manuscript.

## CONFLICT OF INTEREST STATEMENT

The authors declare no competing interests.

## ETHICS STATEMENT

This study was approved by the Human Ethics Committee of Panzhihua Central Hospital (approval no. 20200009). This study was based on the publicly available GWAS summary statistics. Adequate patient consent and ethical approval were obtained from the original studies from which the data for this study were obtained. This study was approved by the Institutional Review Board of Panzhihua Hospital (Panzhihua City, Sichuan, China) and conducted according to the ethical guidelines of the 1975 Declaration of Helsinki and the International Conference on Harmonization Guidelines for Good Clinical Practice.

## CONSENT FOR PUBLICATION

All cases of the report have agreed to consent for publication.

## DECLARATION OF GENERATIVE AI IN SCIENTIFIC WRITING

The authors declare no use of generative artificial intelligence (AI) and AI‐assisted technologies in the writing process.

## PATIENT CONSENT STATEMENT

The authors confirm that patient consent forms have been obtained for this article.

## Supporting information


**Table S1:** Independent pQTLs of CAMK1, BGAT and ACSS1.
**Table S2:** The results of Mendelian randomization analysis in our study.
**Table S3:** The results of sensitivity analysis in our study.
**Table S4:** Baseline characteristics of the study sample.

## Data Availability

The datasets generated and/or analyzed during the current study are available in the GWAS datasets.
